# Digital Referral Patterns for Otolaryngology Subspecialties: A Two-Year Nationwide Analysis in Saudi Arabia

**DOI:** 10.3390/healthcare14172675

**Published:** 2026-08-22

**Authors:** Abdullah A. Alharbi, Meshary S. Binhotan, Ahmad Y. Alqassim, Mohammed A. Muaddi, Abdulaziz Alhosaini, Mohammed S. Arafat, Abdulrahman Aldhabib, Ibrahim Sumaily, Nawfal A. Aljerian

**Affiliations:** 1 Department of Family & Community Medicine, College of Medicine, Jazan University, Jazan 45142, Saudi Arabia; 2 Emergency Medical Services Department, College of Applied Medical Sciences, King Saud bin Abdulaziz University for Health Science, Riyadh 11481, Saudi Arabia; 3 King Abdullah International Medical Research Centre, Riyadh 11481, Saudi Arabia; 4 Medical Referrals Centre, Ministry of Health, Riyadh 12382, Saudi Arabia; 5 Otorhinolaryngology, Head and Neck Surgery Department, King Fahad Central Hospital, Jazan Health Cluster, Jazan 82666, Saudi Arabia

**Keywords:** digital referral systems, E-referral systems, otolaryngology, ENT, public health, Saudi Arabia

## Abstract

**Background**: Otolaryngology conditions significantly influence public health globally, often posing challenges in accessing specialized ear, nose, and throat (ENT) services. Digital referral systems can facilitate access to specialized care while supporting resource allocation. This study analyzed ENT digital referrals through the digital referral system of the Saudi Medical Referrals Centre to describe utilization patterns across different ENT subspecialties. **Methods**: This retrospective cross-sectional study analyzed 50,514 ENT digital referrals across Saudi Arabia from January 2023 to December 2024. Crude population-based rates per 10,000 were calculated using national census data. Cross-tabulation tables were utilized to compare patient demographics, regional variables, and referral characteristics with ENT subspecialties. **Results**: ENT digital referral utilization over two years reached 15.70 per 10,000 population (7.38 in 2023, and 8.32 in 2024). Patients over 60 years showed the highest rates (42.17), followed by children aged 1–7 years (29.90). Females demonstrated a higher crude population-based rate (17.07 vs. 14.83) despite males comprising 57.76% of total referrals. General ENT services represented 59.49% of referrals, audiology services 32.92%, while specialized services showed lower volumes. Routine outpatient referrals predominated at 91.14%, and only 1.3% of total requests required intensive care beds. Most referrals were managed internally (93.75%) with overall acceptance rate reaching 89.41%. **Conclusions**: The findings demonstrate distinct age- and subspecialty-related utilization patterns within Saudi Arabia’s national ENT digital referral system, with the highest demand among older patients and young children. General ENT constituted the majority of referrals, while other specialized services appeared in smaller volumes. The predominantly outpatient, highly accepted, and internally managed referral patterns establish baseline metrics for national ENT subspecialty access. These findings outline nationwide coordination patterns of subspecialty care and support continued investment in digital health infrastructure for specialized care coordination.

## 1. Introduction

Otolaryngology care faces substantial systemic barriers, including fragmented paper-based referral systems, poor adherence to referral criteria, and prolonged waits reaching 328 days [1,2,3]. These delays are particularly problematic for time-sensitive conditions such as cochlear implantation, where optimal outcomes depend on intervention within 12–18 months and critical periods expire by the age of 3 years [4]. Delayed subspecialized ear, nose, and throat (ENT) referrals can prolong symptoms and impair quality of life, with refractory rhinosinusitis patients experiencing an estimated 63 lost productive workdays annually and substantial productivity losses [5]. Geographic and socioeconomic factors compound access challenges, with rural populations facing longer travel distances and provider shortages compared to urban areas [6,7,8,9]. However, coordinated approaches demonstrate reform potential, with direct audiology referral models reducing wait times from 328 days to 13 days while managing 34% of patients without ENT intervention [3,10], highlighting integrated referral systems’ capacity to ensure timely intervention.

The integration of digital technology into healthcare delivery has emerged as a solution to address referral process challenges through digital referral system implementation [11]. Digital referral platforms have demonstrated positive outcomes, including reduced referral processing times and higher completion rates, thereby enhancing specialized care access and patient satisfaction [12,13]. These improvements are crucial, as timely access to specialized care can improve patient outcomes while reducing healthcare system costs [14]. This has led multiple countries to integrate digital referral systems within their national healthcare frameworks [15,16,17]. Efficient referral coordination has become fundamental to healthcare system sustainability and effectiveness, serving as an indicator of healthcare governance capacity in managing complex care pathways [18,19].

The Saudi healthcare system has undergone comprehensive national transformation as part of Saudi Vision 2030, extending beyond infrastructure expansion to include advanced technology integration within the healthcare ecosystem [20]. This transformation focuses on improving healthcare access and modernizing resources, with innovation and e-health services expansion constituting fundamental elements of the strategy aimed at improving care quality [20]. This commitment is evident across various healthcare sectors, including telemedicine and artificial intelligence integration, and the establishment of SEHA Virtual Hospital, the largest virtual hospital in the world, which remotely provides healthcare support to physical hospitals across Saudi Arabia using advanced digital technologies [21,22]. The Saudi digital referral system has also been revamped as part of this transformation [23].

Saudi Arabia has implemented a nationwide digital referral system through the Medical Referrals Centre (MRC) [23]. This system differs from conventional digital referral platforms that typically operate at hospital or regional levels, connecting primary care with specialty services or linking specialized providers [24,25]. MRC functions at a national scale, facilitating referral coordination between secondary, tertiary, and specialized healthcare facilities, while integrating Ministry of Health (MOH) hospitals, governmental hospitals, and the majority of private healthcare providers within a unified digital infrastructure [23]. The platform manages comprehensive referral requests across all medical specialties for all age groups throughout Saudi Arabia’s healthcare regions [23,26,27]. MRC, previously known as Saudi Medical Appointments and Referrals Centre (SMARC), has shown advancements in coordinating and securing access to patients across several specialties including mental health, obstetrics and gynecology, and those requiring intensive care units [23,28,29,30]. However, comprehensive systematic analysis of ENT services utilization patterns within this national digital referral framework remains unexplored. Despite some studies highlighting the utilization of ENT services within the MRC digital platform, these studies aimed to showcase the MRC’s performance in managing digital referrals across different specialties and did not conduct an in-depth analysis of the key aspects of ENT services (e.g., required subspecialties, required bed type, and geographic patterns) [23,26,27]. This represents a significant knowledge gap in understanding how large-scale digital referral systems manage subspecialty ENT care distribution and access patterns.

This study aims to analyze ENT digital referral patterns within Saudi Arabia’s national digital referral system during 2023–2024, using the MRC database. Specifically, the study’s objectives are to: (1) describe ENT subspecialty referral patterns, including patient demographics and regions; (2) analyze referral request characteristics (e.g., required bed type, referral type, and destination) across different ENT subspecialties; and (3) measure referral acceptance outcomes. Collectively, these objectives will establish comprehensive baseline metrics for ENT digital referral patterns across Saudi Arabia, highlighting potential ENT demand and providing insights to inform digital referral policy and planning.

## 2. Materials and Methods

### 2.1. Study Design and Setting

We conducted a nationwide retrospective cross-sectional analysis of ENT digital referrals processed through the MRC digital platform from January 2023 to December 2024. We started data collection and analysis in January 2025 and continued until April 2025. The MRC system coordinates all ENT referrals using the Unified System of Medical Referrals (USMR), providing digital access for hospitals to submit referral requests.

This study analyzed ENT referrals across Saudi Arabia’s healthcare network, administratively organized into five business units (BUs): Central BU (Riyadh and Al-Qassim regions), Eastern BU (Eastern region), Western BU (Makkah, Madinah, and Al-Baha), Northern BU (Al-Jouf, Northern Borders, Tabuk, and Hail), and Southern BU (Asir, Jazan, and Najran) [30].

### 2.2. Data Source and Collection

Data were extracted from the MRC digital referral database, which included 745,680 national referral requests across all specialties, using the referral request as the unit of analysis. Of these, 51,614 ENT referrals were identified and included for eligibility screening to examine the pattern of ENT referrals, while referrals from all other specialties were excluded.

Prior to final data extraction, data validation was performed within the MRC system through systematic quality checks for completeness, consistency, and accuracy across all variables. To identify referral acceptance and avoid duplicate bookings, each referral request was assigned a unique referral code and sent simultaneously to multiple hospitals. Once one hospital accepted the referral, the system automatically rejected the same referral at the remaining hospitals. Conversely, for referrals rejected by all receiving hospitals, only one rejection record was retained in the dataset and coded as rejected. To ensure data accuracy, each patient’s referral pathway was carefully reviewed through a deduplication process.

ENT referrals with incomplete documentation, such as missing key variables (e.g., request type and required bed), were classified as incomplete and excluded from the final analytic dataset. These incomplete referrals represented less than 3% of ENT referrals. After exclusions, the final ENT referral sample included 50,514 referrals, which were included in the final analysis of this study.

While the ENT data used in this study were derived from a large-scale national referral project encompassing all medical and surgical specialties, they provide novel ENT-specific findings. Prior research utilizing the MRC database has provided a broad overview of referral activity across specialties but has not offered an in-depth examination of ENT subspecialties [23,26,27]. In contrast, the present study focuses specifically on ENT subspecialties, covering general otolaryngology, audiology, speech disorders, head and neck surgery, and cochlear implantation. This focused approach provides novel findings on the demographic distribution, geographical variation, bed requirements, and referral outcomes associated with ENT referrals, thereby supporting more precise, specialty-specific planning and policy development.

### 2.3. MRC Referral Process

**Figure 1:**
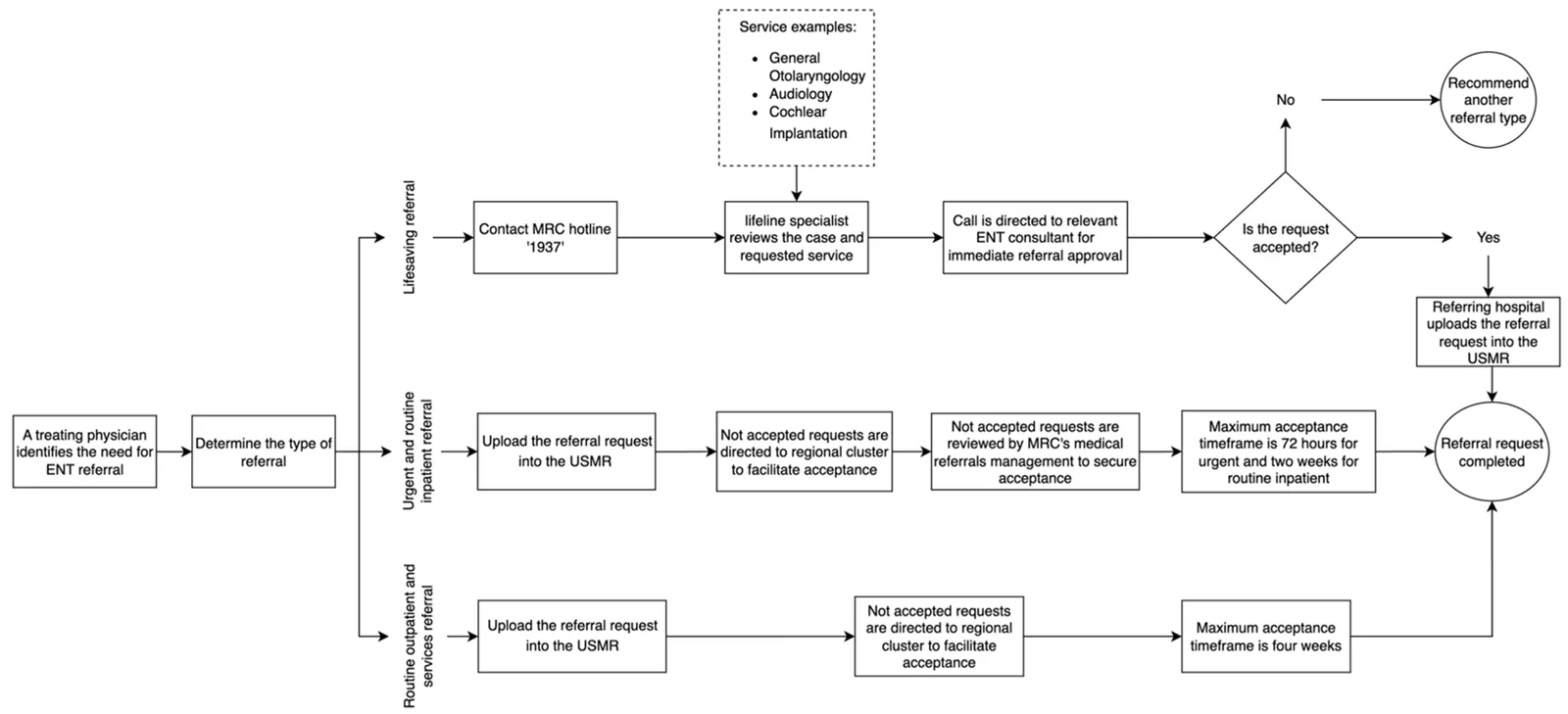
The process of ENT digital referral requests through the Saudi Medical Referrals Centre (MRC) digital referral system.

As shown in Figure 1, the MRC system categorizes ENT referrals based on clinical urgency: routine outpatient department (OPD) referrals, routine inpatient referrals, urgent referrals, services referrals for specific procedures, and lifesaving referrals. Each category follows defined time standards for acceptance, established by the MRC: 72 h for urgent referrals, two weeks for routine inpatient referrals, and four weeks for routine outpatient and services referrals. Lifesaving referrals are expedited through simultaneous hotline contact (1937) for immediate acceptance.

Physicians initiate digital referrals by selecting appropriate ENT specialty/subspecialties (general otolaryngology, audiology, speech disorders, head and neck surgery, or cochlear implantation), referral types, and bed types. Referrals are distributed to suitable facilities where required recourses are available. Receiving facilities review clinical documentation and make acceptance or rejection decisions through the platform.

### 2.4. Variables and Measurements

Demographic variables included patient age (<1 year, 1–7 years, 8–17 years, 18–39 years, 40–60 years, and >60 years) and sex. Geographic variables included BU of origin to assess regional utilization. We calculated referral rates per 10,000 population using updated population estimates for Saudi Arabia [31].

Referral characteristics included ENT subspecialty, referral type (OPD, inpatient, urgent—for cases requiring a referral within 72 h based on the treating physician’s assessment, services—for temporary referrals to conduct specific procedures, and lifesaving—for cases requiring immediate approval according to the treating physician’s assessment), bed type (OPD no bed, ward bed, intensive care unit, pediatric intensive care unit, neonatal intensive care unit, cardiac care unit), destination facility (internal referrals within the same administrative region versus external referrals between different regions), and referral outcome (accepted or rejected). We analyzed referral volumes across 2023 and 2024 to identify temporal trends.

### 2.5. Statistical Analysis

Descriptive statistics included frequencies and percentages for categorical variables. Crude referral rate per 10,000 population over the two-year study period (2023–2024) was calculated using the latest demographic data from the Saudi General Authority for Statistics, published in 2022 [31], and stratified by age group, sex, and BUs, applying the formula: (referrals in stratum ÷ population of the same stratum) × 10,000. Annual ENT digital referral rates for 2023 and 2024 were calculated using the national population denominator, and 95% confidence intervals were estimated using Poisson-based methods. Cross-tabulation tables were utilized to compare patient demographics, regional variables (classified by BUs), and referral characteristics (referral type, bed type, referral destination, acceptance status, and request year) with ENT subspecialties. Referral acceptance rates were computed as the proportion of accepted referrals relative to total referral requests, with stratification applied across ENT subspecialties. To identify factors independently associated with acceptance, a multivariable binary logistic regression (outcome: accepted vs. rejected) was fitted, including age, sex, BU, subspecialty, referral type, bed type, referral direction, and year. Adjusted odds ratios (aORs) with 95% CIs are reported. The analysis was conducted using Stata Statistical Software version 16 (StataCorp, 2021, College Station, TX, USA).

### 2.6. Ethical Considerations

This study received ethical approval from the Institutional Review Board of the Saudi Ministry of Health (protocol code 23-77-E, approved on 20 September 2023). The dataset was fully de-identified with no personal identifiers. Individual informed consent requirements were waived due to the retrospective nature and use of de-identified data. All procedures adhered to Declaration of Helsinki principles.

## 3. Results

A total of 50,514 ENT digital referral requests were submitted through the MRC during 2023–2024. Age-, sex-, and region-specific patterns, presented as 2-year cumulative rates against the same denominator, are shown in Table 1. Rates were highest among patients over 60 years and children aged 1–7 years, and lowest in infancy and among adults aged 18–39. Crude rates were higher among females than males despite males comprising the larger share of referrals in absolute numbers. Rates were also highest in the Northern and Southern BUs and lowest in the Central and Eastern units. The annual referral rate rose from 7.38 per 10,000 population in 2023 to 8.32 per 10,000 in 2024 (Table 2).

General otolaryngology and audiology accounted for the large majority of referrals, while speech disorders, head and neck surgery, and cochlear implantation together represented a small minority (Table 3).

Subspecialty composition varied by age: speech disorder referrals were concentrated in children, head and neck surgery referrals in adults, and cochlear implantation referrals predominantly in early childhood. Male referral rates exceeded female rates only for speech disorders. Regional differences in subspecialty composition were also observed, most notably a low volume of cochlear implantation referrals from the Southern business unit (Table 4).

Most referrals specified no inpatient bed and were classified as routine outpatient; referrals requiring critical care beds were uncommon, together accounting for 1.3% of the total. Outpatient predominance was near-universal for audiology and speech disorders, while general otolaryngology and head and neck surgery included larger proportions of ward, urgent, and lifesaving referrals. Most referrals were managed within the referring administrative region; external referral was more frequent for head and neck surgery and cochlear implantation than for other subspecialties. Overall, 89.41% of referrals were accepted, with lower acceptance for speech disorders and cochlear implantation than for the remaining subspecialties (Table 5).

Table 6 presents multivariable logistic regression results, revealing significant associations. The Eastern BU referrals had markedly higher odds than the Central reference (aOR 7.49, 95% CI 5.79–9.69), and internal referrals were around four times higher than external referrals (aOR 4.02, 95% CI 3.65–4.43). Referrals in 2024 had lower odds than in 2023 (aOR 0.41, 95% CI 0.39–0.44), while urgent and lifesaving referrals had the highest odds of acceptance. Also, acceptance odds were higher for ward and critical care bed requests than for outpatient requests, and lower for speech disorders than for general ENT. Sex and most age groups were not independently associated with acceptance.

## 4. Discussion

This study analyzed ENT digital referral patterns within Saudi Arabia’s nationwide MRC system, examining 50,514 referrals to characterize subspecialty distribution and utilization patterns. Key findings revealed an overall two-year cumulative referral rate of 15.70 per 10,000 population with peak utilization among patients over 60 years (42.17) and young children (1–7 years: 29.90). The acceptance rate reached 89.41%, with 93.75% of all referrals managed internally. General otolaryngology and audiology dominated referral volumes, while specialized services showed distinct utilization patterns with very low volume. Regional variations were observed with Northern and Southern BUs showing higher rates (35.47 and 32.26 respectively) compared to Central and Eastern regions. These findings provide baseline data on ENT service utilization through a large-scale digital referral system and describe nationwide coordination patterns of subspecialty care.

The observed age-specific referral patterns align with established ENT epidemiological distributions. Highest utilization rates in patients over 60 years reflect the high prevalence of age-related hearing loss (presbycusis), which affects approximately 63% of adults over 70 and more than 80% of those over 80, potentially explaining elevated audiology referral rates in this population [32]. General ENT high referral rates in elderly patients likely correspond to increased prevalence of epistaxis, chronic rhinosinusitis, and age-related surgical considerations [33,34]. Peak rates in children aged 1–7 years align with typical incidence periods for adenotonsillar hypertrophy, recurrent otitis media, and developmental speech delays [35]. The concentration of speech disorder referrals in this age group may reflect critical developmental windows for language acquisition [36]. Crude population-based referral rates of females exceeded males overall, except for speech disorders where male predominance aligns with higher developmental speech delay prevalence in males [37,38]. These patterns could suggest appropriate capture of high-risk populations during critical developmental and disease periods.

The data show that most general ENT and audiology referrals were completed within the referring region, whereas head and neck surgery and cochlear implantation had the highest external referral proportions. This pattern is plausibly consistent with established delivery models in which common conditions are managed locally while complex, low-volume procedures are concentrated in expert centers [39,40], although this interpretation was not directly examined and requires confirmation in future studies.

The data also demonstrate higher crude population-based referral rates in the Northern and Southern BUs. However, this cross-sectional analysis of referral records cannot determine the underlying causes of this variation. Plausible, non-mutually exclusive explanations include regional differences in ENT disease burden, population age structure, extent of MRC platform utilization, case identification and referral thresholds, and healthcare-seeking behavior. Variations in health service utilization across regions [41] and between physicians [42] is well documented internationally, and utilization patterns in Saudi Arabia have similarly been linked to region of residence and socio-demographic characteristics [43]. These explanations remain hypotheses warranting future studies integrating regional epidemiological and clinical data. What the data directly show is a predominance of intra-regional referrals, with a small proportion of external referrals directed to centers providing complex interventions. The MOH’s facilitated referral system—covering transportation and accommodation for inter-regional transfers [44], with short domestic flights (approximately 1.5–2 h to the central BU)—supports accessible inter-regional care when needed.

The referral patterns were predominantly outpatient-based, most pronounced in audiology, speech disorders, and cochlear implantation services which typically involve diagnostic evaluations. General ENT and head and neck surgery showed lower outpatient proportions, reflecting surgical intervention requirements. The majority of referrals were routine, with minimal critical care representation, consistent with typical ENT subspecialty consultation severity profiles [45]. Service referrals provided temporary access to specialized diagnostics while maintaining care continuity. This outpatient emphasis aligns with established ENT practice patterns where most subspecialty consultations are appropriately managed in ambulatory settings, associated with reduced healthcare costs compared to inpatient delivery [24,45,46]. These patterns could reflect how required services differ across ENT subspecialties.

Referral acceptance showed significant associations with region, referral direction, year, bed type, and subspecialty. The Eastern BU recorded the highest acceptance odds, consistent with earlier Saudi digital referral findings across all specialties (aOR 2.39) [47]. The Eastern BU has successfully implemented the new Model of Care as part of the healthcare transformation program in Saudi Arabia, advancing the quality and accessibility of healthcare services and promoting the prevention of health risks [48]. Sociodemographic and disease burden differences among regions may also have influenced referral acceptance. Referrals in year 2024 had lower odds of acceptance compared to those in 2023, which may be attributable to differences in the severity of referred cases or growing maturity of the referral system, with more severe conditions increasingly prioritized over time. Internal referrals were associated with a higher likelihood of acceptance compared to external ones, aligning with previous Saudi digital referral findings (aOR 0.68) [27]. Possible explanations include the eligibility criteria for external referrals, the time sensitivity of cases and clinical stability required for inter-facility transfer [49], and the availability of requested services internally, which may reduce the need for external services. Urgent and lifesaving referrals also showed the highest odds of acceptance, potentially reflecting the prioritization of critical cases, as early interventions can improve patient outcomes [50,51]. However, further research examining the actual factors influencing referral acceptance across multiple dimensions (e.g., operational, clinical, and contextual factors) is warranted to enhance our understanding and inform policymaking.

The high acceptance and internal management rates observed in this study across different ENT subspecialities could reflect nationwide access to ENT services coordinated through the digital referral system. Relatively lower acceptance rates in cochlear implantation and speech disorders might be attributed to specific clinical eligibility requirements, including minimum age criteria, mandatory hearing aid trial periods, and comprehensive preoperative assessments [52]. More broadly, these favorable outcomes are consistent with evidence that digital referral systems improve communication and reduce wait times compared to paper-based systems [24]. Studies indicate that standardized referral criteria and digital documentation reduce rejections due to incomplete information while maintaining clinical standards [53,54]. These findings illustrate the large volume of referrals coordinated by the MRC digital platform across multiple subspecialties and geographic regions, comparable to large-scale international implementations such as the Mid and North Coast Health Pathways program [55].

### 4.1. Policy Implications

The findings of this study can provide insights into several policy implications. Coordinating high-volume digital referral requests over two years with observed high acceptance rate may indicate potential for the scalability of centralized digital referral platforms. Regional variations highlight the importance of population-based healthcare planning that accounts for demographic differences when allocating subspecialty resources. Predominant outpatient delivery suggests potential for cost-effective care models, while the concentration of complex procedures in specialized centers aligns with centralized excellence models for high-complexity interventions. These patterns could inform evidence-based policy decisions regarding subspecialty workforce distribution, infrastructure investment priorities, and service delivery optimization.

### 4.2. Future Directions

This study suggests several directions for future research. Longitudinal studies could assess the MRC digital referral system’s impact on patient health outcomes and care quality over time. Investigating operational metrics such as patient satisfaction and timeliness (e.g., time from submission to acceptance, and waiting time), alongside referral status (cancellation, completion, and unattended appointments), would enhance understanding of system efficiency and user experience. Qualitative analysis of the views of referring and receiving physicians could shed light on the challenges and facilitators of the digital referral system, improving both user experience and system performance. Comparative analyses across national healthcare systems using digital referral platforms would help identify best practices and optimization strategies applicable to similar contexts. Deeper investigation into rejected digital referrals would clarify the underlying reasons for rejection, providing insights for developing guidelines to improve digital referral system quality. Further studies could also examine adjusted referral-rate variations across demographic groups, regions, and time; multivariable predictors of referral acceptance; factors associated with internal versus external referrals; and multilevel associations between patient-, facility-, and region-level factors (including resource allocation) and referral outcomes, with appropriate adjustment for clustering by hospital, region, or patient. Exploring the relationship between centralized excellence models for complex procedures and clinical outcomes could further inform resource allocation strategies. Finally, assessing the cost-effectiveness of implementing an ENT referral pathway within a nationwide digital referral system would provide valuable economic insights for healthcare policy.

### 4.3. Strengths

This study’s strengths include a comprehensive analysis of a large national digital referral dataset, including multiple ENT subspecialties across diverse geographic regions, providing insights into the description of ENT digital referrals and service demand at scale. The two-year data collection period captures temporal trends, while crude population-based rate calculations enable regional comparisons. The inclusion of multiple outcome measures, including acceptance rates, referral patterns, and care delivery settings, provides multidimensional view of ENT referral activity across different ENT subspecialties.

### 4.4. Limitations

This study has some limitations that warrant consideration. Reliance on secondary data and the retrospective design restricted the inclusion of additional variables, such as clinical data, that would assess the impact of digital referrals on patient clinical outcomes. While the study analyzed acceptance rates, operational data such as referral timeliness (e.g., time from submission to acceptance, and waiting time) and patient satisfaction were not included. Lastly, the referral rate per 10,000 population for the study period (2023 and 2024) was measured relying on a single 2022 denominator from the Saudi General Authority for Statistics. This 2022 census, however, represents the most recent published population data offering the level of detail required for our analysis (e.g., population stratified by region).

## 5. Conclusions

This study examined the utilization patterns of ENT digital referrals through Saudi Arabia’s national digital platform. Distinctive patterns were observed including higher crude population-based rates among females, concentration in general ENT and audiology services, while other specialized services like cochlear implantation showed the lowest utilization. Peak referral rates occurred in patients over 60 years and in young children. The acceptance rate reached 89.41%, and most cases were managed intra-regionally, while complex subspecialty services were directed to specialized centers. Referral acceptance showed significant associations with several factors, including Eastern BU and internal referrals. These findings establish baseline metrics for ENT subspecialty access through digital referral infrastructure and illustrate the volume of referrals managed through a centralized digital referral network within large healthcare systems. The results support the viability of digital health transformation initiatives, aligned with achieving the 2030 health transformation vision, and suggest potential applicability for similar integrated healthcare systems internationally. Further research examining clinical outcomes, operational performance metrics, and cost-effectiveness of digital referral systems would provide deeper insights into the broader impact of such models.

## Figures and Tables

**Table 1:** Demographic and Geographic Characteristics of ENT Digital Referrals Across Saudi Arabia (2-year cumulative).

Characteristics	Frequency (N)	Percentage (%)	Population (N)	Rate per 10,000 Population
Total	50,514	100.00	32,175,224	15.70
Age				
<1 year	157	0.31	495,438	3.17
1–7 years	11,255	22.28	3,764,106	29.90
8–17 years	9102	18.02	4,989,917	18.24
18–39 years	14,197	28.11	14,553,614	9.75
40–60 years	10,109	20.01	7,021,943	14.40
>60 years	5694	11.27	1,350,206	42.17
Sex				
Male	29,178	57.76	19,678,595	14.83
Female	21,336	42.24	12,496,629	17.07
Referring Business Unit (BU)				
Central BU	9420	18.65	9,927,927	9.49
Eastern BU	4407	8.72	5,125,254	8.60
Western BU	14,486	28.68	10,498,620	13.80
Northern BU	9229	18.27	2,601,841	35.47
Southern BU	12,972	25.68	4,021,582	32.26

Note: The rate per 10,000 population was calculated using (referrals in stratum ÷ population of the same stratum) × 10,000, representing the 2-year cumulative rate for 2023–2024. Population statistics were adopted from the Saudi General Authority for Statistics, using the latest published population data from 2022 [31].

**Table 2:** Annual ENT Digital Referral Rates, Saudi Arabia, 2023–2024.

Characteristics	Frequency (N)	Percentage (%)	Population (N)	Rate per 10,000 Population (95% CI)
Total	50,514	100.00	32,175,224	15.70 (15.56–15.84)
Request Year				
2023	23,753	47.02	32,175,224	7.38 (7.29–7.48)
2024	26,761	52.98	32,175,224	8.32 (8.22–8.42)

Note: The total rate per 10,000 population represents the cumulative 2-year referral rate (2023–2024), whereas the year-specific rates represent annual referral rates. Population statistics were adopted from the Saudi General Authority for Statistics, using the latest published population data from 2022 [31]. CI: Confidence Interval.

**Table 3:** ENT Digital Referrals Metrics: Subspecialties, Directions, and Acceptance Outcomes Across Saudi Arabia (2-year cumulative).

Characteristics	Frequency (N)	Percentage (%)
Total	50,514	100.00
ENT subspecialities		
General Otolaryngology (ENT)	30,049	59.49
Audiology	16,631	32.92
Speech Disorders	2963	5.87
Head and Neck Surgery	706	1.40
Cochlear Implantation	165	0.33
Referral Destination		
External	3158	6.25
Internal	47,356	93.75
Referral Decision		
Accept	45,167	89.41
Reject	5347	10.59

**Table 4:** Distribution of Age Groups, Sex, and Business Units by Requested ENT Subspecialties in Saudi Arabia (2-year cumulative).

Characteristics	General Otolaryngology (ENT)		Audiology		Speech Disorders		Head and Neck Surgery		Cochlear Implantation		Total	
	N (%)	Rate/10,000	N (%)	Rate/10,000	N (%)	Rate/10,000	N (%)	Rate/10,000	N (%)	Rate/10,000	N (%)	Rate/10,000
Age												
<1 year	81 (0.27)	1.63	75 (0.45)	1.51	0 (0.00)	0.00	1 (0.14)	0.02	0 (0.00)	0.00	157 (0.31)	3.17
1–7 years	5868 (19.53)	15.59	3406 (20.48)	9.05	1886 (63.65)	5.01	17 (2.41)	0.05	78 (47.27)	0.21	11,255 (22.28)	29.90
8–17 years	5880 (19.57)	11.78	2443 (14.69)	4.90	701 (23.66)	1.40	57 (8.07)	0.11	21 (12.73)	0.04	9102 (18.02)	18.24
18–39 years	9869 (32.84)	6.78	3808 (22.90)	2.62	203 (6.85)	0.14	283 (40.08)	0.19	34 (20.61)	0.02	14,197 (28.11)	9.75
40–60	5672 (18.88)	8.08	4061 (24.42)	5.78	124 (4.18)	0.18	230 (32.58)	0.33	22 (13.33)	0.03	10,109 (20.01)	14.40
>60 years	2679 (8.92)	19.84	2838 (17.06)	21.02	49 (1.65)	0.36	118 (16.71)	0.87	10 (6.06)	0.07	5694 (11.27)	42.17
Sex												
Male	17,455 (58.09)	8.87	9135 (54.93)	4.64	2106 (71.08)	1.07	392 (55.52)	0.20	90 (54.55)	0.05	29,178 (57.76)	14.83
Female	12,594 (41.91)	10.08	7496 (45.07)	6.00	857 (28.92)	0.69	314 (44.48)	0.25	75 (45.45)	0.06	21,336 (42.24)	17.07
Business Unit (BU)												
Central BU	6322 (21.04)	6.37	2546 (15.31)	2.56	338 (11.41)	0.34	173 (24.50)	0.17	41 (24.85)	0.04	9420 (18.65)	9.49
Eastern BU	3095 (10.30)	6.04	953 (5.73)	1.86	258 (8.71)	0.50	56 (7.93)	0.11	45 (27.27)	0.09	4407 (8.72)	8.60
Western BU	8815 (29.34)	8.40	4316 (25.95)	4.11	1125 (37.97)	1.07	198 (28.05)	0.19	32 (19.39)	0.03	14,486 (28.68)	13.80
Northern BU	4594 (15.29)	17.66	3630 (21.83)	13.95	880 (29.70)	3.38	85 (12.04)	0.33	40 (24.24)	0.15	9229 (18.27)	35.47
Southern BU	7223 (24.04)	17.96	5186 (31.18)	12.90	362 (12.22)	0.90	194 (27.48)	0.48	7 (4.24)	0.02	12,972 (25.68)	32.26

Note: The rate per 10,000 population was calculated using (referrals in stratum ÷ population of the same stratum) × 10,000, representing the 2-year cumulative rate for 2023–2024. Population statistics were adopted from the Saudi General Authority for Statistics, using the latest published population data from 2022 [31].

**Table 5:** Characteristics of Digital Referrals by Requested ENT Subspecialties Across Saudi Arabia, 2023–2024.

Characteristics	General Otolaryngology (ENT) N (%)	Audiology N (%)	Speech Disorders N (%)	Head and Neck Surgery N (%)	Cochlear Implantation N (%)	Total N (%)
Total	30,049 (100.00)	16,631 (100.00)	2963 (100.00)	706 (100.00)	165 (100.00)	50,514 (100.00)
Bed Type						
OPD	25,889 (86.16)	16,572 (99.65)	2952 (99.63)	612 (86.69)	160 (96.97)	46,185 (91.43)
Ward bed	3528 (11.74)	53 (0.32)	10 (0.34)	74 (10.48)	5 (3.03)	3670 (7.27)
ICU	372 (1.24)	1 (0.01)	1 (0.03)	17 (2.41)	0 (0.00)	391 (0.77)
PICU	147 (0.49)	1 (0.01)	0 (0.00)	1 (0.14)	0 (0.00)	149 (0.29)
NICU	89 (0.30)	4 (0.02)	0 (0.00)	2 (0.28)	0 (0.00)	95 (0.19)
CCU	24 (0.08)	0 (0.00)	0 (0.00)	0 (0.00)	0 (0.00)	24 (0.05)
Referral Type						
Routine OPD	25,770 (85.76)	16,555 (99.54)	2947 (99.46)	608 (86.12)	160 (96.97)	46,040 (91.14)
Routine inpatient	1877 (6.25)	42 (0.25)	9 (0.30)	27 (3.82)	1 (0.61)	1956 (3.87)
Urgent	1863 (6.20)	27 (0.16)	2 (0.07)	60 (8.50)	1 (0.61)	1953 (3.87)
Services	407 (1.35)	7 (0.04)	5 (0.17)	0 (0.00)	3 (1.82)	422 (0.84)
Life-saving	132 (0.44)	0 (0.00)	0 (0.00)	11 (1.56)	0 (0.00)	143 (0.28)
Referral Destination						
External	1950 (6.49)	851 (5.12)	146 (4.93)	176 (24.93)	35 (21.21)	3158 (6.25)
Internal	28,099 (93.51)	15,780 (94.88)	2817 (95.07)	530 (75.07)	130 (78.79)	47,356 (93.75)
Referral Decision						
Accept	27,023 (89.93)	14,908 (89.64)	2468 (83.29)	631 (89.38)	137 (83.03)	45,167 (89.41)
Reject	3026 (10.07)	1723 (10.36)	495 (16.71)	75 (10.62)	28 (16.97)	5347 (10.59)
Request Year						
2023	14,338 (47.72)	7736 (46.52)	1320 (44.55)	278 (39.38)	81 (49.09)	23,753 (47.02)
2024	15,711 (52.25)	8895 (53.48)	1643 (55.45)	428 (60.62)	84 (50.91)	26,761 (52.98)

OPD: Outpatient Department, ICU: Intensive Care Unit, PICU: Pediatric Intensive Care Unit, NICU: Neonatal Intensive Care Unit, CCU: Cardiac Care Unit, N (%): Number (Percentage), Services: Referrals for specific diagnostic procedures or therapeutic interventions with return to originating facility for continued care.

**Table 6:** Multivariable Logistic Regression Analysis of Factors Associated with ENT Referral Acceptance.

Variable	aOR	95% CI	*p*-Value
Age group			
18–<40 years (ref)	1.00	—	—
<1 year	0.55	0.37–0.82	0.004
1–<8 years	0.89	0.81–0.96	0.005
8–<18 years	1.09	0.99–1.20	0.071
40–60 years	0.99	0.91–1.08	0.830
>60 years	0.93	0.84–1.04	0.185
Sex			
Male (ref)	1.00	—	—
Female	1.02	0.96–1.08	0.494
Business Unit			
Central (ref)	1.00	—	—
Eastern	7.49	5.79–9.69	<0.001
Western	0.75	0.69–0.82	<0.001
Northern	1.20	1.08–1.32	<0.001
Southern	1.15	1.05–1.25	0.004
Year of referral			
2023 (ref)	1.00	—	—
2024	0.41	0.39–0.44	<0.001
Bed requirement			
OPD (ref)	1.00	—	—
Ward bed	3.41	1.98–5.85	<0.001
ICU/CCU	3.53	1.81–6.89	<0.001
PICU/NICU	1.55	0.79–3.05	0.200
Referral type			
Urgent/lifesaving (ref)	1.00	—	—
Routine inpatient	0.40	0.29–0.54	<0.001
Routine outpatient	0.79	0.46–1.35	0.384
Services	0.31	0.17–0.59	<0.001
Referral direction			
External referral (ref)	1.00	—	—
Internal referral	4.02	3.65–4.43	<0.001
ENT sub-specialty			
General Otolaryngology (ENT) (ref)	1.00	—	—
Audiology	1.07	1.00–1.14	0.054
Speech disorders	0.63	0.56–0.71	<0.001
Head and neck surgery	1.38	1.07–1.78	0.012

aOR = adjusted odds ratio; CI = confidence interval; ICU = intensive care unit; CCU = coronary care unit; PICU = pediatric intensive care unit; NICU = neonatal intensive care unit.

## Data Availability

The data used in this study contains health information subject to national data protection laws, thus restricting public sharing. Aggregated or anonymized data may be made available upon reasonable request to the corresponding author and after obtaining the necessary regulatory approvals.

## Abbreviations

The following abbreviations are used in this manuscript:

ENT	Ear, Nose, and Throat
E-referral	Electronic Referral
MRC	Medical Referrals Centre
USMR	Unified System of Medical Referrals
BU	Business Unit
OPD	Outpatient Department
ICU	Intensive Care Unit
PICU	Pediatric Intensive Care Unit
NICU	Neonatal Intensive Care Unit
CCU	Cardiac Care Unit
MOH	Ministry of Health
